# Communication Between Traditional Medical Practitioners and Western Medical Professionals

**DOI:** 10.3389/fsoc.2019.00037

**Published:** 2019-06-21

**Authors:** Fanuel Lampiao, Joseph Chisaka, Carol Clements

**Affiliations:** ^1^Africa Centre of Excellence in Public Health and Herbal Medicine, College of Medicine, University of Malawi, Blantyre, Malawi; ^2^Biomedical Sciences Department, College of Medicine, University of Malawi, Blantyre, Malawi; ^3^Institute of Pharmacy and Biomedical Sciences, University of Strathclyde, Glasgow, United Kingdom

**Keywords:** traditional healers, biomedical practitioners, herbal medicine, collaboration, traditional medicine

## Abstract

The high burden of disease in Malawi is exacerbated by a lack of healthcare professionals, and the inaccessibility of healthcare services to many Malawians, due to geographical and financial barriers. The World Health Organization commends the contribution that traditional and complementary medicine could make to achieve such coverage through its integration into health systems. This study aimed to evaluate the barriers that exist between traditional healers and biomedical practitioners for them to collaborate with each other. Semi-structured interviews were conducted with traditional healers and biomedical practitioners. Results showed that the two groups were willing to collaborate with each other, but to differing degrees. Traditional healers were more enthusiastic than biomedical practitioners, who had several reservations about traditional healers, and placed certain conditions on prospective collaboration. While traditional healers clearly had confidence in biomedical practitioners' competencies and respect for their practice, biomedical practitioners lacked trust in traditional healers and would not refer patients to them due to several reservations, such as the lack of scientific basis for traditional medicine. This study points out barriers that affects collaboration between traditional healers and biomedical practitioners and it suggests possible solutions.

## Introduction

Integration of traditional and complementary medicine into healthcare systems is an increasingly popular concept, not only in Africa, but across the globe (Bodeker and Kronenberg, 2002). In many low- and middle-income countries such as Malawi, resource-poor healthcare services struggle to manage the significant disease burden that exists (Msyamboza et al., 2011). Such countries are increasingly facing a recognized double burden of communicable and non-communicable diseases (World Health Organization, 2015). The pre-existing absolute poverty, along with its associated risk factors, continues to contribute to this burden of morbidity and mortality.

Traditional medicine (TM) is defined by the World Health Organization (WHO) as:

“*the sum total of the knowledge, skill, and practices based on the theories, beliefs, and experiences indigenous to different cultures, whether explicable or not, used in the maintenance of health as well as in the prevention, diagnosis, improvement or treatment of physical and mental illness”* (World Health Organization, 2013).

TM remains a main source of healthcare to many in Africa, as it has been throughout the continent's history (World Health Organization, 2013). About 80% of Malawi's population are thought to seek treatment from traditional healers (THs) (Simwaka et al., 2007), and a number of reasons are suggested for their popularity. Firstly, THs share their patients' culture, and share beliefs about disease (Simwaka et al., 2007). Secondly, THs have a holistic approach, taking into account a patient's social life, relationships, external environment and spiritual wellbeing (Simwaka et al., 2007). One study found THs to be considered more respectful and approachable than their biomedical counterparts (Munthali et al., 2014). Another reason for their popularity is their accessibility: many practice in rural villages, so people need not travel long distances to see them (Courtright et al., 2000; Munthali et al., 2014), and they are often cheaper than conventional medicine. For some, TM is a last resort after failure of conventional medicine to cure them (Munthali et al., 2014). Currently in Malawi people have plural health seeking behavior using both traditional and western medicine.

In Malawi, there are several traditional healer associations, overseen by the Malawi Traditional Healers Umbrella Organization (MTHUO), through which the Malawi Ministry of Health (MoH) communicates with and supports these associations. Even though, there is such structure, very little collaboration takes place between THs and biomedical practitioners (BPs). This study aimed to explore the barriers to collaboration between TH and BPs.

## Methodology

### Study Design

An exploratory qualitative study design was chosen. A qualitative approach was thought suitable for the aims, which involved eliciting perceptions, opinions and attitudes of participants, and attempting to understand the reasons behind these. Very little is known about collaboration between THs and BPs in Malawi. This study therefore explored this subject further, in particular, in the setting of the Blantyre and Mulanje district, Malawi.

This study is based on the “*Structuration Model of Collaboration”* (SMoC), described by D'Amour et al. (2008). The model suggests four dimensions, themselves comprising 10 indicators to evaluate collective action in healthcare delivery. These dimensions comprise two relational dimensions, concerning the interaction between partners of a collaboration, and organizational dimensions, concerning the direction and implementation of collaboration. This model was chosen due to its suitability for application to healthcare settings and to integration of traditional healers and healthcare services, shown by its use in Chung et al. (2012).

### Study Location

The study took place in Blantyre and Mulanje Districts, Malawi. This study was ethically approved by the College of Medicine Research and Ethics Committee, an institutional review board of the College of Medicine, University of Malawi.

### Study Population and Data Collection

Maximum variation sampling was used to recruit participants with as much variation as possible, but who were relevant to the study and able to provide the desired data. BPs from a wide range of specialties and THs who were herbalists with different amounts of experience, and of both genders were sought. A total of 12 THs, 6 males and 6 females were recruited while 11 BPs 6 males and 5 females were recruited in this study. Eight THs were from the rural area and four from urban setting while seven BPs were from urban and four from rural areas. Semi-structured interviews with topic guide were conducted to obtain data.

### Data Analysis

Qualitative data analysis was used in this study. Manual deductive and inductive thematic analysis was carried out on the interview transcriptions. Data sets were initially analyzed separately. After familiarization with the data, initial codes were produced, which were largely latent, rather than semantic, i.e., the data was interpreted to a degree to generate codes by which to classify it. Themes were generated by grouping several codes together under broader headings.

## Results

The results were divided into three themes namely, goals, vision of collaboration, mutual knowledge and respect. These themes were divided into subthemes.

## Goals

The dimension on which this theme is based concerns the goals, motives and allegiances possessed by collaborative partners, their respective visions of collaboration, and the degree of overlap of each of these.

### Participants' Goals and Motivations to Practice

Goals and motivations of THs' practice included treating or helping people, promoting a healthy society, and promoting their own business. One TH was quoted saying:

“*What motivates me is whenever I treat someone, and then they get healed, so whenever they are happy, and they are cured completely, I feel better, that's what motivates me to go on.”*

BPs goals and motivations included helping and treating patients and promoting a health community.

### Perceived Shared Goals

All THs confirmed having a sense of alliance with BPs, and thought both had the same aims, which were to treat patients and promote a healthy society.

Seven BPs reported they thought they had a shared aim with THs, which was to treat patients, though they acknowledged differences between their practices. Four BPs did not think they shared any goals.

### Patient-Centered Orientation vs. Other Allegiances

Four THs described involving their patients in the decision about treatment, by offering treatment options, or giving a specially requested treatment. One TH described treating his patients with respect and consideration, and mentioned this as an area for improvement for BPs.

All of the THs described referring patients they could not manage to the hospital. Examples of such cases were anemia, conditions requiring operations, cholera, dehydration, malaria, and women in labor. Some THs said they would refer patients with possible malaria or HIV, because the hospital had the necessary diagnostic equipment, though one of these healers thought that malaria could actually be better treated by THs.

All BPs described the importance of patient-centered care, and the effort they make to provide this. Reasons they gave for this were patient satisfaction and patient compliance. One BP suggested that THs' allegiance to their own business prevents their referral of patients (and arguably, their patient-centeredness).

### Vision of Collaboration

Four THs said they would like to undertake training with BPs, to teach each other about their own professions. In particular, one TH said he would like to learn how to preserve drugs, as they do in the hospital, and one suggested THs and BPs could work in the same hospital. Four THs wanted a mutual referral system, whereby each group sends patients they can't treat to the other. These THs suggested BPs should refer to them illnesses that they are not able to treat especially those that arise due to bewitchment.

### All BMPs Proposed Research Into the Remedies Used by THs

“*But probably, we can try to explore the medicine which they use, and then see if this works. But that has to undergo vigorous research, testing before we come to a conclusion.”*

Two BPs suggested training THs how to better manage some cases, and when to refer patients to the hospital.

## Vision of Collaboration

This theme comprises the subthemes willingness, motivations, and perceived barriers.

### Willingness

All of the THs expressed enthusiasm and willingness to collaborate with BPs. There was a general consensus that collaboration was extremely important as this TH explains:

“*I am very pleased with this initiative. I think it's something in the right direction. Bringing together people from the medical side and from the traditional healer side to discuss and know what the two groups are doing.”*

All BPs expressed some interest in collaboration, to varying degrees. One BP felt collaboration was very important, two appeared open to it, and one admitted that potential existed for collaboration, but was against THs treating patients.

“*I collaborate with them in research. We must, we should, I think…that's a big need.”*

### Motivations

Some of the THs expressed extreme gratitude at being invited to participate in this study, considering their invitation as recognition of their profession and a step toward collaboration.

For BPs the motivations for collaboration included the possibility of identifying new drugs, educating THs to improve their practice and limit harm caused by malpractice and delayed referrals, and reaching patients not accessing mainstream healthcare. Three BPs felt that THs' role in society could not be disregarded due to their popularity and accessibility.

### Perceived Barriers

Six THs said they did not perceive any barriers to collaboration, while six said a barrier was a lack of a platform for communication as stated by one TH:

“*I think we lack the capability to interact with doctors face to face, like asking each other questions on how they perceive a certain disease, on how they can treat a certain disease, on how they can handle some diseases. We don't have a platform where we can discuss the issues.”*

For BPs suggested barriers to collaboration included mutual mistrust, and THs' lack of education and inability to understand medical teaching. One BP discussed THs' fear of exploitation and loss of business.

## Mutual Knowledge and Respect

This theme regards the knowledge collaborative partners have of one another in both personal and professional capacities, and to what extent they respect the other and their competencies.

### Mutual Acquaintanceship

Five THs reported to have friends who worked in the hospital, and one healer described how she became friends with a nurse by helping cure her. Nine BPs described knowing THs from their villages while growing up and two BPs said they did not know any THs personally.

All THs claimed to know about how BPs practiced. Specific examples they gave of BPs practices included: giving prescriptions; doing cesarean sections; diagnosing diseases with tools and machines; implementing public health interventions; giving drips or blood transfusions; and, giving health promotion advice. Four THs said that BPs could not treat magical or superstitious diseases. When asked if BPs understood TM, most THs said that they did, while only one thought not.

All BPs had some knowledge of THs. One BP knew there are different types of TH, including spiritual healers and herbalists, though three knew that some may have superstitious or spiritual practices. One BP described THs' practices, including prescribing oral remedies or making incisions. Anemia, dermatological symptoms, and snake bites were suggested as conditions THs may be able to treat, and several BPs discussed a traditional remedy could induce labor contractions.

THs said they had learned about BPs through several means, including the media, personal experience, and school. BPs had learned about THs through exposure while growing up, rural placements at medical school, and patient referrals from THs.

### Trust

All THs said that BPs were important or useful. One TH said she thought BPs were more important than THs. Furthermore, one TH said that BPs were more advanced than THs, and four said that BPs were important because they were able to treat diseases that THs couldn't.

Two THs said that they thought BPs looked down on them, considering them to be less competent.

One BP reported that while herbal medicine could be useful, he did not approve of any other forms of traditional healing. Three BPs did not think that traditional healing could treat any condition better than conventional medicine, though one suggested that THs may be better equipped to deal with certain conditions than state hospitals.

All THs claimed to refer patients they could not treat to the hospital. One TH said if he could not treat someone in a given time period, he would then refer them. When asked if BPs referred patients to them, three THs said that they did. Two of these healers said they were referred patients thought to have a magic disease, and one said that he was referred patients with diabetes and sexually transmitted diseases. Two THs were never referred patients. One TH said BPs' code of conduct did not allow referral to THs.

No BPs had ever referred to THs. Two BPs hadn't been referred patients from THs, and two others said THs had referred patients to them with cancer, HIV-related conditions, or women in labor.

## Discussion

This study aimed to investigate the willingness of traditional healers and biomedical practitioners in the Blantyre and Mulanje districts, Malawi, to collaborate with each other, and to estimate the current and future levels of collaboration, using the SMoC. The findings were presented under the theme headings: goals, vision of collaboration, mutual knowledge and respect.

The enthusiasm for collaboration expressed by THs and the mixed, but overall positive response from BPs, is in keeping with literature findings (Sorsdahl et al., 2010; Bryman, 2016). It is interesting to review the motivations for both participant groups to collaborate; while at first glance, their specific motivations may seem to differ, ultimately both groups wish to improve patient care. THs wish to increase their competence, and ensure that patients see the most appropriate health worker, while BPs wish to discover new drugs, educate THs to prevent harmful practices and limit delayed referrals, and to identify patients not accessing mainstream healthcare. These motivations are also documented in the literature (Okeke et al., 2006). In their study into perceptions of inter-professional collaboration, Ødegård and Strype (2009) found motivation to be one of four most important aspects to collaboration, and the most important individual characteristic, perceived by healthcare professionals.

Our findings show THs and BPs do share goals and motivations in their practice, which are to treat patients and promote a healthy society. While no BPs mentioned business or financial gain as a personal motivation unlike THs, its relevance to private medicine was noted.

Despite a BP's assertion that THs avoid referring patients to protect their income and reputation, all THs claimed to refer cases they couldn't handle to the hospital, suggesting allegiance to their patients. THs were previously instructed by the MoH to refer certain cases to the hospital, therefore THs may have claimed to refer patients to avoid criticism. It also appeared that the BPs main allegiance was to their patients, though one BP discussed how patient-centered care suffered due to a lack of time and resources in state health facilities. None of the BPs interviewed were known to practice private medicine, though it would be interesting to know how this may affect a BP's allegiance. This study has shown that both parties have allegiance to patients, and although they may hold other allegiances, (e.g., their business) this is a positive sign of collaborative potential.

The differences in visions of collaborations held by both parties reflect their patient-allegiance and reservations. The findings are consistent with literature findings, concerning BPs' disapproval of a mutual referral system, and the desire of THs to learn more about western medicine (Peltzer and Khoza, 2002).

It was found that both THs and BPs had a reasonable understanding of the general principles, approach, and scope of each other's practice. Given the high exposure to THs described by BPs when growing up in a culture such as Malawi's, it is not surprising that they had a good understanding of TM. Several other studies have also shown BPs to have some knowledge of TM (Awodele, 2012; Appelbaum Belisle et al., 2015; Nemutandani et al., 2016). It is worth considering the possibility that proximity to urban societies with western medicine, and higher exposure to mainstream media (through which two healers in this study learned about hospital medicine), might increase THs' knowledge of western healthcare, and possibly influence their practice and referral behavior. Several studies evaluate THs existing knowledge about particular “western” diseases, typically in the context of collaborative programmes for conditions such as HIV/AIDS (Furin, 2011), TB (Heinzerling, 2005), and malaria (Okeke et al., 2006), however comparison with this study is difficult, due to the general nature of this study's enquiry on this subject.

The trust and respect of BPs demonstrated by THs is found elsewhere in the literature, where THs are reported to refer their patients willingly (Peltzer et al., 2006; Viney et al., 2014; Keikelame and Swartz, 2015; Van Rooyen et al., 2015). Similarly, the reservations held by BPs about referring to THs are also described several times (Burnett et al., 1999; Peltzer and Khoza, 2002; Okeke et al., 2006; Campbell-Hall et al., 2010; Musyimi et al., 2016; Nemutandani et al., 2016). The lack of trust on the part of BPs makes collaboration a one-way referral system and education of THs, very difficult. In order for collaboration to be made possible, their reservations must be addressed, possibly through regulation and standardization of THs' practices, and research into their medicines.

## Conclusion

This study sought to investigate the willingness of THs and BPs in Blantyre and Mulanje Districts, Malawi, to collaborate with each other, and to estimate the current and future levels of collaboration. The THs and BPs who participated in this study were overall willing to collaborate with each other, though the THs were clearly more enthusiastic about collaboration, and the BPs held several reservations. THs and BPs were found to share goals and motivations in their medical or healing practices, though their visions of collaboration differed according to their perceptions and trust of each other. THs and BPs had a reasonable understanding of each other, but while THs demonstrated trust of BPs, reciprocal trust from BPs was lacking. There is growing evidence about effectiveness or use of TM and Complementary Therapies, we propose that this should be taught in Medical Schools so that BPs can appreciate THs.

## Ethics Statement

This study was carried out in accordance with the recommendations of College of Medicine Research and Ethics Committee (COMREC) with written informed consent from all subjects. All subjects gave written informed consent in accordance with the Declaration of Helsinki. The protocol was approved by the COMREC.

## Author Contributions

All authors listed have made a substantial, direct and intellectual contribution to the work, and approved it for publication.

### Conflict of Interest Statement

The authors declare that the research was conducted in the absence of any commercial or financial relationships that could be construed as a potential conflict of interest.

## References

[B1] Appelbaum Belisle H. Hennink M. Ordóñez C. E. John S. Ngubane-Joye E. Hampton J.. (2015). Concurrent use of traditional medicine and ART: perspectives of patients, providers and traditional healers in Durban, South Africa. Glob. Public. Health 10, 71–87. 10.1080/17441692.2014.967709 25346069 PMC4262581

[B2] Awodele O. (2012). Doctors' attitudes towards the use of herbal medicine in Lagos, Nigeria. J. Herb. Med. 2, 16–22. 10.1016/j.hermed.2012.02.002

[B3] Bodeker G. Kronenberg F. (2002). A public health agenda for traditional, complementary, and alternative medicine. Am. J. Public Health 92, 1582–1591. 10.2105/AJPH.92.10.1582 12356597 PMC3221447

[B4] Bryman A. (2016). Social Research Methods, 5th Edn. New York, NY: Oxford University Press.

[B5] Burnett A. Baggaley R. Ndovi-Macmillan M. Sulwe J. Hang'omba B. Bennett J. (1999). Caring for people with HIV in Zambia: are traditional healers and formal health workers willing to work together? AIDS Care 11, 481–491. 10.1080/09540129947875 10533542

[B6] Campbell-Hall V. Petersen I. Bhana A. Mjadu S. Hosegood V. Flisher A. J. (2010). Collaboration between traditional practitioners and primary health care staff in South Africa: developing a workable partnership for community mental health services. Transcult. Psychiatry. 47, 610–628. 10.1177/1363461510383459 20940271

[B7] Chung V.C. Ma P. H. Hong L.C. Griffiths S. M. (2012). Organizational determinants of interprofessional collaboration in integrative health care: systematic review of qualitative studies. PLoS ONE 7:50022. 10.1371/journal.pone.0050022 23209634 PMC3510174

[B8] Courtright P. Chirambo M. Lewallen S. Chana H. Kanjaloti S. (2000). Collaboration with African Traditional Healers for the Prevention of Blindness. Singapore: World Scientific Publishing Co. Pte. Ltd.

[B9] D'Amour D. Goulet L. Labadie J. F. Martín-Rodriguez L. S. Pineault R. (2008). A model and typology of collaboration between professionals in healthcare organizations. BMC Health Serv. Res. 8:188. 10.1186/1472-6963-8-188 18803881 PMC2563002

[B10] Furin J. (2011). The role of traditional healers in community-based HIV care in rural Lesotho. J. Commun. Health 36, 849–856. 10.1007/s10900-011-9385-3 21374087

[B11] Heinzerling L. M. (2005). Attitudes of traditional healers towards Western medicine in rural Cameroon. Trop. Doct. 35, 161–164. 10.1258/0049475054620653 16105343

[B12] Keikelame M. J. Swartz L. (2015). A thing full of stories: Traditional healers' explanations of epilepsy and perspectives on collaboration with biomedical health care in Cape Town. Transcult. Psychiatry. 52, 659–680. 10.1177/1363461515571626 25680366 PMC4552613

[B13] Msyamboza K. P. Ngwira B. Dzowela T. Mvula C. Kathyola D. Harries A. D.. (2011). The burden of selected chronic non-communicable diseases and their risk factors in malawi: Nationwide STEPS survey. PLoS ONE 6, 6–11. 10.1371/journal.pone.0020316 21629735 PMC3100352

[B14] Munthali A.C. Mannah H. MacLachlan M. Swartz L. Makupe C.M. Chilimampunga C. (2014). Non-use of formal health services in Malawi: perceptions from non-users. Malawi. Med. J. 26, 126–132. 26167263 PMC4325348

[B15] Musyimi C. W. Mutiso V. N. Nandoya E. S. Ndetei D. M. (2016). Forming a joint dialogue among faith healers, traditional healers and formal health workers in mental health in a Kenyan setting: towards common grounds. J. Ethnobiol. Ethnomed. 12, 4. 10.1186/s13002-015-0075-6 26742992 PMC4705751

[B16] Nemutandani S. M. Hendricks S. J. Mulaudzi M. F. (2016). Perceptions and experiences of allopathic health practitioners on collaboration with traditional health practitioners in post-apartheid South Africa. Afr. J. Prm. Health Care Fam. Med. 8:e1-8. 10.4102/phcfm.v8i2.1007 27380856 PMC4926717

[B17] Ødegård A. Strype J. (2009). Perceptions of interprofessional collaboration within child mental health care in Norway. J. Interprof. Care 23, 286–296. 10.1080/13561820902739981 19387908

[B18] Okeke T. A. Okafor H. U. Uzochukwu B. S. C. (2006). Traditional healers in nigeria: perception of cause, treatment and referral practices for severe malaria. J. Biosoc. Sci. 38, 491–500. 10.1017/S002193200502660X 16762086

[B19] Peltzer K. Khoza L. (2002). Attitudes and knowledge of nurse practitioners towards traditional healing, faith healing and complementary medicine in the Northern Province of South Africa. Curationis 25, 30–40. 10.4102/curationis.v25i2.749

[B20] Peltzer K. Mngqundaniso N. Petros G. (2006). A controlled study of an HIV/AIDS/STI/TB intervention with traditional healers in KwaZulu-Natal, South Africa. AIDS Behav. 10, 683–690. 10.1007/s10461-006-9110-x 16715347

[B21] Simwaka A. Peltzer K. Maluwa-Banda D. (2007). Indigenous healing practices in Malawi. J. Psychol. Afr. 17, 155–162. 10.1080/14330237.2007.10820162

[B22] Sorsdahl K. Stein D. J. Flisher A. J. (2010). Traditional healer attitudes and beliefs regarding referral of the mentally ill to Western doctors in South Africa. Transcult. Psychiatry 47, 591–609. 10.1177/1363461510383330 20940270

[B23] Van Rooyen D. Prestorius B. Tembani N. M. ten Ham W. (2015). Allopathic and traditional health practitioners' collaboration. Curationis 38:1495. 10.4102/curationis.v38i2.1495 26244463 PMC6092702

[B24] Viney K. Johnson P. Tagaro M. Fanai S. Linh N. N. Kelly P.. (2014). Traditional healers and the potential for collaboration with the national tuberculosis programme in Vanuatu: results from a mixed methods study. BMC Publ. Health. 14, 393. 10.1186/1471-2458-14-393 24758174 PMC4011835

[B25] World Health Organization (2013). Traditional Medicine Strategy: 2014-2023. Geneva. Available online at: http://apps.who.int/iris/bitstream/10665/92455/1/9789241506090_eng.pdf (accessed December 10, 2016).

[B26] World Health Organization (2015). Malawi: WHO Statistical Profile. Available online at: http://who.int/gho/mortality_burden_disease/en/ (accessed October 30, 2016).

